# The Relative Biological Effectiveness for Carbon and Oxygen Ion Beams Using the Raster-Scanning Technique in Hepatocellular Carcinoma Cell Lines

**DOI:** 10.1371/journal.pone.0113591

**Published:** 2014-12-02

**Authors:** Daniel Habermehl, Katarina Ilicic, Sarah Dehne, Stefan Rieken, Lena Orschiedt, Stephan Brons, Thomas Haberer, Klaus-Josef Weber, Jürgen Debus, Stephanie E. Combs

**Affiliations:** 1 Department of Radiation Oncology, Im Neuenheimer Feld 400, 69120, Heidelberg, Germany; 2 Heidelberg Ion Beam Therapy Center (HIT), Im Neuenheimer Feld 450, 69120, Heidelberg, Germany; 3 Department of Radiation Oncology, Klinikum rechts der Isar, Technische Universität München, Isma-ninger Str. 22, 81675, Munich, Germany; University of Navarra School of Medicine and Center for Applied Medical Research (CIMA), Spain

## Abstract

**Background:**

Aim of this study was to evaluate the relative biological effectiveness (RBE) of carbon (12C) and oxygen ion (16O)-irradiation applied in the raster-scanning technique at the Heidelberg Ion beam Therapy center (HIT) based on clonogenic survival in hepatocellular carcinoma cell lines compared to photon irradiation.

**Methods:**

Four human HCC lines Hep3B, PLC, HepG2 and HUH7 were irradiated with photons, 12C and 16O using a customized experimental setting at HIT for in-vitro trials. Cells were irradiated with increasing physical photon single doses of 0, 2, 4 and 6 Gy and heavy ionsingle doses of 0, 0.125, 0.5, 1, 2, 3 Gy (12C and 16O). SOBP-penetration depth and extension was 35 mm +/−4 mm and 36 mm +/−5 mm for carbon ions and oxygen ions respectively. Mean energy level and mean linear energy transfer (LET) were 130 MeV/u and 112 keV/um for 12C, and 154 MeV/u and 146 keV/um for 16O. Clonogenic survival was computated and realtive biological effectiveness (RBE) values were defined.

**Results:**

For all cell lines and both particle modalities α- and β-values were determined. As expected, α-values were significantly higher for 12C and 16O than for photons, reflecting a steeper decline of the initial slope of the survival curves for high-LET beams. RBE-values were in the range of 2.1–3.3 and 1.9–3.1 for 12C and 16O, respectively.

**Conclusion:**

Both irradiation with 12C and 16O using the rasterscanning technique leads to an enhanced RBE in HCC cell lines. No relevant differences between achieved RBE-values for 12C and 16O were found. Results of this work will further influence biological-adapted treatment planning for HCC patients that will undergo particle therapy with 12C or 16O.

## Introduction

Treatment options for patients with advanced hepatocellular carcinoma (HCC) are limited and prognosis still remains poor [1]. Potential curative treatments such as surgery, liver transplantation or locoregional therapies including radiofrequency ablation or chemoembolization can only be administered in early-stage disease [2]. Conventional radiotherapy (RT) has been applied for treatment of HCC in the past with only modest results. Better results with high local control rates can be achieved by using modern high-precision radiation techniques such as stereotactic body radiotherapy (SBRT) with large single doses [3], [4]. Dose tolerance of surrounding normal liver tissue is a limiting factor for the application of high local doses [5], [6]. However new radiation modalities such as particle therapy offer promising physical and biological characteristics and present therefore an alternative treatment option for patients with HCC [7]–[9]. Ion beams are characterized by an inverse dose-depth profile with a maximal dose deposition in a predefined depth in tissues (Bragg-peak) while the surrounding organs at risk can be spared as far as possible. A further advantage of this treatment modality compared to photons is the high-LET (Linear Energy Transmission) character of heavy ion beams such as carbon (12C) and oxygen (16O) ions [10]. High-LET irradiation exerts a higher relative biological effectiveness (RBE) in tumour cells which therefore can overcome relative radio-resistance induced by hypoxia through induction of clustered DNA double-strand breaks [11], [12]. Oxygen ions (16O) have a higher mass compared to carbon ions (12C) which can therefore theoretically be translated into a slight biological superiority with the advantage of achieving higher RBE values in irradiated tissues.

Aim of this study was to evaluate in-vitro effects of carbon and oxygen ion-irradiation applied in the raster-scanning technique on clonogenic survival in hepatocellular carcinoma cell lines compared to photon radiotherapy. Results of this work will further influence biological-adapted treatment planning for HCC patients at HIT.

## Materials and Methods

### Cells and cell culture

All cell lines were obtained from the American Type Culture Collection (ATCC, Manassas, VA, USA). The three human HCC lines Hep3B, PLC and HUH7 were grown in DMEM (Dubelco’s modified Eagle Medium; Biochrom, Berlin, Germany), HepG2 cells were maintained in RPMI 1640 medium (Biochrom, Berlin, Germany). Both media were supplemented with 10% heat-inactivated fetal bovine serum (FBS) (Gibco, Life Technologies, Vienna, Austria) and 1% penicillin-streptomycin (Sigma-Aldrich GmbH, Munich, Germany). Cells were stored lying flat in 175 cm^2^ tissue plastic flasks in an incubator at 37°C in humidified air with 5% CO_2_ and passaged weekly. Mycoplasma screening was performed regularly by a dedicated laboratory.

### Photon irradiation

Photon irradiation was performed with a biological cabinet X-ray irradiator (XRAD 320 Precision X-ray Inc., N. Bradford, CT) at single doses of 2, 4, 6 and 8 Gy. Irradiation was performed at room temperature.

### Carbon and oxygen ion irradiation

Carbon and oxygen ion treatment was performed at the Heidelberg Ion-Beam Therapy Center (HIT) using the raster-scanning technique. Single doses of 0.125, 0.5, 1, 2 and 3 Gy were delivered with an extended Spread-Out Bragg peak (SOBP) that was adjusted using a 3.5-cm acrylic shield and positioning cell monolayers in the middle of the SOBP. SOBP-penetration depth and extension was 35 mm +/−4 mm and 36 mm +/−5 mm for carbon ions and oxygen ions respectively. Mean energy level and mean linear energy transfer (LET) were 130 MeV/u and 112 keV/um for carbon ions, and 154 MeV/u and 146 keV/um for oxygen ions. Irradiation was performed at room temperature. Experiments were done as previously described by our group [13].

### Colony forming assay

In order to generate reliable results every experiment was performed in triplets two or three times at independent days. Clonogenics were performed as previously described by our group [13], [14]. Firstly, a defined and increasing amount of cells, adjusted to the increasing doses of irradiation were seeded into 25 cm^2^ flasks filled with medium and incubated for 24 h before irradiation. Secondly, after irradiation cells were incubated 5 to 14 days depending on cell line and its individual extent of forming colonies. Thirdly, cell fixation was done using 4 ml of 70% ethanol followed by 5 ml of methylene blue for 10 min. Finally, colony counting was performed under the microscope with a threshold of minimum 50 cells per colony. From the determined surviving fractions the plating efficiency and clonogenic survival were calculated. These results were used to generate survival curves, to define α- and β-parameters and to calculate RBE-values. SigmaPlots’ (Systat Software GmbH, Erkrath, Germany) non-linear least-squares regression option was used to fit the linear-quadratic expression [-ln(S) = α*D+β*D^2^] to the resulting averaged survival fractions after normalizing plating efficiencies to the untreated samples (S is the number of surviving cell following a dose of D, and α and β are the respective sensitivity coefficients).

## Results

### Photon irradiation

**Figure 1:**
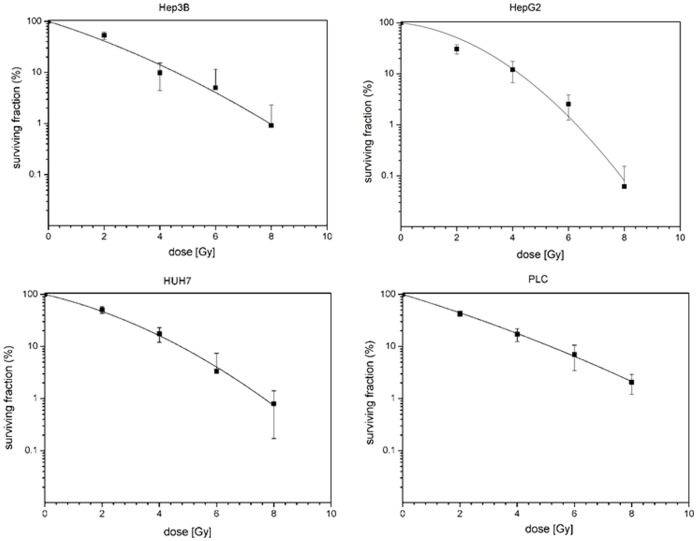
Fitted survival curves after photon-irradiation of HepG2, Hep3B, HuH7 and PLC cell lines determined by colony forming assay. Applied doses were 0, 2, 4, 6 and 8 Gy. All survival curves showed a dose-dependent decrease of clonogenic cells.

In a first set of experiments HCC cell lines HepG2, HuH7, Hep3B and PLC were irradiated with increasing doses of 0, 2, 4, 6 and 8 Gy. Cell lines showed a dose-dependent suppression of clonogenic survival as can be seen by typical shoulder-shaped curves (figure 1). The surviving fractions determined were used to perform linear quadratic fits and calculate clonogenic survival curves for each cell line in order to compare the effectiveness of photon and 12C as well as 16O irradiation.

### 12C and 16O irradiation

**Figure 2:**
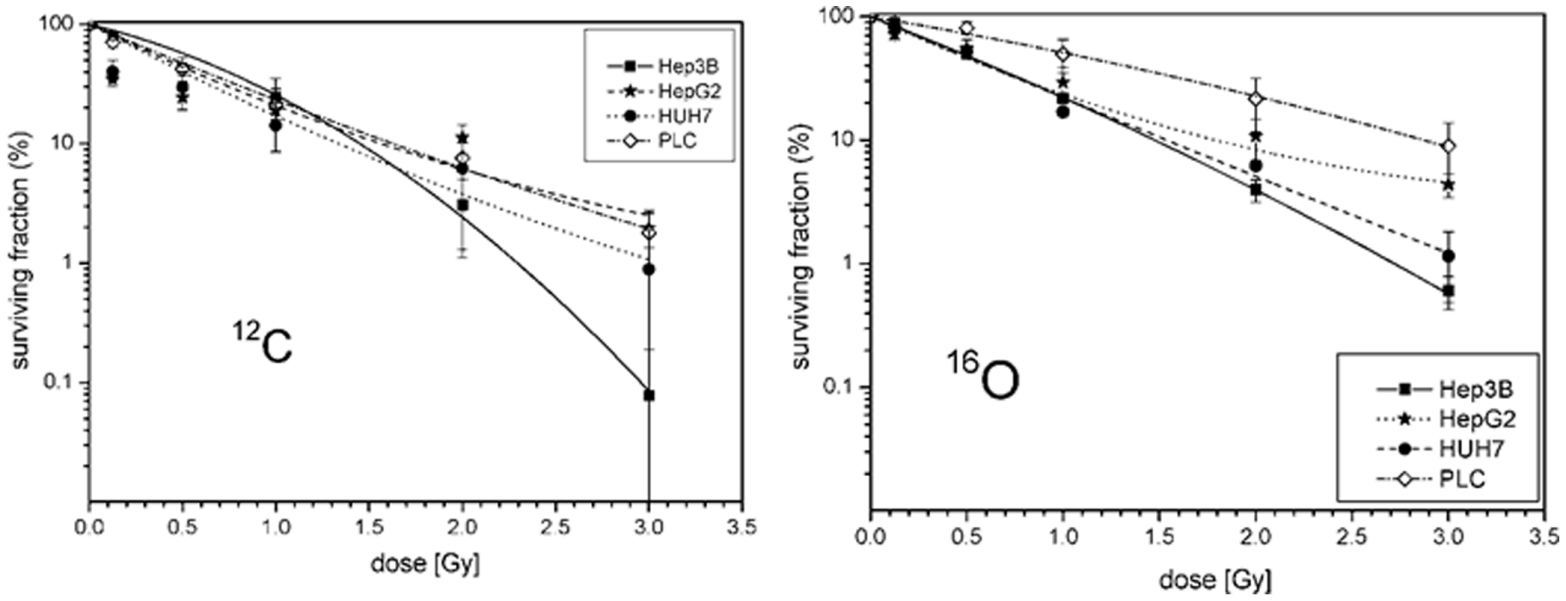
Fitted survival curves after 12C- (on the left) and 16O-irradiation (on the right) of HepG2, Hep3B, HuH7 and PLC cells lines determined by colony forming assay. Applied doses were 0, 0.125, 1, 2 and 3 Gy for both radiation modalities. All survival curves showed a dose-dependent decrease of clonogenic cells.

In a second set of experiments the above mentioned cell lines were treated with 12C and 16O ion beams and again clonogenic survival was assessed by a colony forming assay. Applied physical doses were 0, 0.125, 0.5, 1, 2 and 3 Gy dependent on cell line and particle modality. All cell lines showed a dose-dependent suppression of clonogenic survival (figure 2).

**Figure 3:**
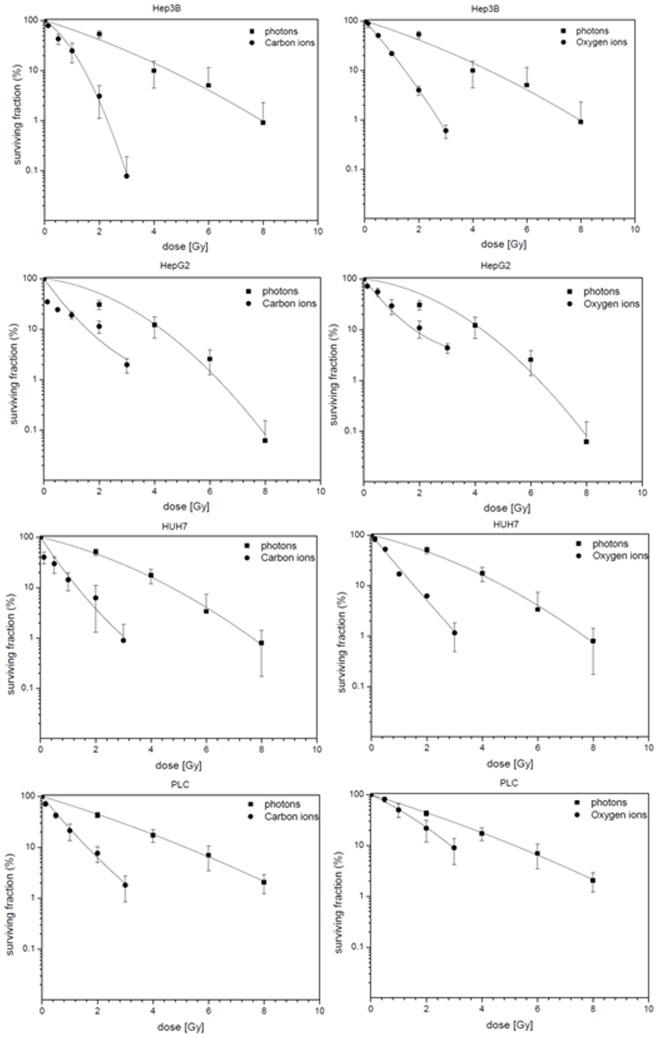
Fitted survival curves after photon, 12C- and 16O-irradiation of Hep3B, HepG2, HuH7 and PLC cell lines determined by colony forming assay (shown in descending order). Applied photon doses were 0, 2, 4, 6 and 8 Gy and applied ion doses for 12C and O16 were 0, 0.125, 1, 2 and 3 Gy. Survival curves of HCC cell lines after photon and 12C irradiation are shown on the left side, survival curves after photon and O16 irradiation are shown on the right side.

In comparison to the photon-RT derived survival data of the tested cell lines, survival fractions for 12C and 16O treated cells were even lower (figure 3). Therefore, both particle beam modalities 12C and 16O showed an enhanced efficacy towards clonogenic cell death induction than low-LET irradiation with photons.

### RBE calculation and determination of α- and β-parameters

For all cell lines and both particle modalities α- and β-values were determined and are presented in table 1. As expected, α-values were significantly higher for 12C and 16O than for photons, reflecting a steeper decline of the initial slope of the survival curves for high-LET beams. RBE values are calculated at 10% cell survival (RBE_10_) and consistent for all 12C- and 16O-treated cell lines (table 2). Resulting RBE-values were in the range of 2.1–3.3 and 1.9–3.1 for 12C and 16O, respectively. Obviously there are no significant differences between achieved RBE-values for 12C and 16O. Only for PLC cells the RBE value for 16O was exceptionally low compared to the other cell lines.

**Table 1:** Calculated α- and β-values for HepG2, Hep3B, HuH7 and PLC cells after irradiation with photons, 12C and 16O**.**

		HepG2	Hep3B	HuH7	PLC
**photons**	α	0.1482	0.3966	0.2973	0.3817
	β	0.0927	0.02301	0.03963	0.01244
**12C**	α	1.733	0.8659	1.892	1.531
	β	–0.1685	0.4962	–0.1272	–0.07204
**16O**	α	1.65	1.398	1.527	0.6025
	β	–0.20857	0.1028	–0.01935	0.06914

RBE Relative Biological Effectiveness, ph photons.

**Table 2:** RBE values determined for 12C- and 16O-irradiation of HepG2, Hep3B, HuH7, and PLC cells.

	HepG2	RBE	Hep3B	RBE	HuH7	RBE	PLC	RBE
**photons**	4.2769		4.19983		4.6062		5.30342	
**12C**	2.04784	2.08849	1.43932	2.9179	1.40869	3.2698	1.7277	3.0696
**16O**	2.10514	2.03165	1.465	2.86678	1.51044	3.0496	2.85986	1.8544

RBE Relative Biological Effectiveness, ph photons.

## Discussion

Charged particle beams with carbon or oxygen ions applied using the raster-scanning technique at HIT lead to high RBE values in four tested HCC cell lines. Clonogenic cell survival was dose-dependent and detectable in all examined cell lines. Resulting RBE-values (RBE_10_, determined at 10%-survival) were comparable for 12C and 16O and in the range of 2–3. Based on these data future adoptions and corrections in the context of biological optimized particle therapy of HCC patients at HIT will be possible with the aim of a more exact computation of RBE-tables in the treatment planning environment.

Our work represents the first experimental dataset with 12C- and 16O-ion beams performed at HIT with the active raster-scanning method in HCC cell lines. In general, data on in-vitro RBE-evaluation of heavy ion beams are sparse, especially for 16O. Recently, our group reported RBE-datasets for 12C-irradiation of pancreatic cancer and glioblastoma cell lines and examined the effect of additional anti-tumorigenic substances [13]–[16].

Previously, an Indian group examined the influence of several charged particle beams (including 16O, 12C and lithium ions, 7LI) compared to Cobalt irradiation (^60^Co) in V79 Chinese hamster cells [17]. In their setup Chinese hamster cells were exposed to high-LET 16O-beams (625 keV/m) with doses ranging from 0–9.83 Gy. Cell survival, micronuclei formation, chromosomal aberrations and apoptosis-induction were studied. Finally, the dose response curves revealed that 7Li-beam was most effective in cell killing as well as inducing other nuclear damages followed by 12C, 16O and ^60^Co, in that order. The presented results are basically in accordance with our data regarding higher RBE-values for heavy ion beams. But, in contrast to our results, 12C had a higher impact on cell survival than 16O. One explanation can be the different experimental setup. The Indian group used 16O beams with a different energy and the examined cells are non-cancerous fibroblasts, so finally different biological effects may result.

Ofuchi and colleagues determined RBE-values for hepatoma cell lines (HLE and HLF) after photon and 12C irradiation using the premature chromosome condensation (PCC) technique and colony formation [18]. Hereby, carbon ion beams of 13 and 76 keV/micron were compared to x-rays and lead to RBE-values of 1.10–1.24 and 2.57–2.59, respectively. These results are comparable to our findings regarding RBE values in HCC cell lines, even when the experimental setup and used cell lines are slightly differing. With a focus on cell death mechanisms, a Chinese group recently measured survivin expression in HepG2 cells after low- (x-rays) and high-LET (12C) irradiation. Survivin is an important protein involved in the inhibition of apoptosis (IAP, inhibitor of apoptosis proteins), mainly expressed in a wide variety of tumor cells. The group generated evidence of a differential expression pattern of survivin after RT with both modalities. Carbon ion beams increased survivin expression and a more pronounced G2/M-cell cycle arrest than photon-RT up to 4 Gy, with higher doses leading to a further decrease of its expression. The authors therefore conclude that an anti-apoptotic function of survivin may not be evident, possibly because of a serious damage caused by densely ionizing 12C-RT.

A further important aspect of our work is the possible integration of our data into biologically individualized dose calculation algorithms for the treatment planning procedure for HCC patients. Currently the phase-I/−II clinical trial PROMETHEUS is recruiting HCC patients for a four fraction protocol of increasing doses from 10 to 15 Gy(RBE) [9], [19]. Until now treatment planning is performed with *Syngo PT Planning* developed by Siemens Oncology Care Systems (OCS, Erlangen, Germany) including biologic plan optimization, originally based on the Local Effect Model (LEM) developed by GSI (Gesellschaft für Schwerionenforschung) [20]. Finally, the system is designed for RBE-computation for different tissue types and selected endpoints. An integration of tumor-specific radiobiological parameters (e.g. α/β-values) will help to further improve the dose calculation via the TPS (Treatment Planning System) and thus optimize patient treatment.

Future improvements may also incorporate LET-painting in particle therapy treatment planning systems for tumor regions that are hypoxic and therefore more radio-resistant. For this purpose, Bassler and colleagues recently came up with strategies to overcome the relative radio-resistance of hypoxic tumor regions with LET-painting using 12C and 16O [10]. They could show that ions heavier than 12C may be necessary to overcome hypoxic target regions sufficiently. Finally, they conclude that a 16O boost would be promising to target hypoxic structures of 1–4 cm^3^ in size. Together with the results of a recent meta-analysis on the prognostic relevance of tumor hypoxia in HCC patients, these findings have to be considered in TPS dose calculation based on in-vitro derived radiobiological parameters to optimize particle RT for future HCC patients [21].

The acquired data will be valuable for biologically adapted treatment planning in clinical trials on carbon ion therapy of hepatocellular carcinoma such as our running clinical PROMETHEUS trial (hypofractionated carbon ion therapy for HCC). The presented results represent the biological fundamentals of the clinical application for charged particle treatment using the raster-scanning technique.
