# Correction: The Relative Biological Effectiveness for Carbon and Oxygen Ion Beams Using the Raster-Scanning Technique in Hepatocellular Carcinoma Cell Lines

**DOI:** 10.1371/journal.pone.0117960

**Published:** 2015-02-03

**Authors:** The PLOS ONE Staff

The affiliation for the second author is incorrect. Katarina Ilicic is not affiliated with #1 but with #3 Department of Radiation Oncology, Klinikum rechts der Isar, Technische Universität München, Isma-ninger Str. 22, 81675, Munich, Germany.

## References

[1] Habermehl D, Ilicic K, Dehne S, Rieken S, Orschiedt L, et al. (2014) The Relative Biological Effectiveness for Carbon and Oxygen Ion Beams Using the Raster-Scanning Technique in Hepatocellular Carcinoma Cell Lines. PLoS ONE 9(12): e113591 doi:10.1371/journal.pone.0113591 25460352 10.1371/journal.pone.0113591 PMC4252049

